# Challenges in the implementation of an electronic surveillance system in a resource-limited setting: Alerta, in Peru

**DOI:** 10.1186/1753-6561-2-s3-s4

**Published:** 2008-11-14

**Authors:** Giselle Soto, Roger V Araujo-Castillo, Joan Neyra, Miguel Fernandez, Carlos Leturia, Carmen C Mundaca, David L Blazes

**Affiliations:** 1 Emerging Infections Program, U.S. Naval Medical Research Center Detachment (NMRCD), Lima, Peru; 2 Centro Medico Naval, Lima, Peru; 3 Dirección de Epidemiologia del Ejército, Lima, Peru

## Abstract

**Background:**

Infectious disease surveillance is a primary public health function in resource-limited settings. In 2003, an electronic disease surveillance system (Alerta) was established in the Peruvian Navy with support from the U.S. Naval Medical Research Center Detachment (NMRCD). Many challenges arose during the implementation process, and a variety of solutions were applied. The purpose of this paper is to identify and discuss these issues.

**Methods:**

This is a retrospective description of the Alerta implementation. After a thoughtful evaluation according to the Centers for Disease Control and Prevention (CDC) guidelines, the main challenges to implementation were identified and solutions were devised in the context of a resource-limited setting, Peru.

**Results:**

After four years of operation, we have identified a number of challenges in implementing and operating this electronic disease surveillance system. These can be divided into the following categories: (1) issues with personnel and stakeholders; (2) issues with resources in a developing setting; (3) issues with processes involved in the collection of data and operation of the system; and (4) issues with organization at the central hub. Some of the challenges are unique to resource-limited settings, but many are applicable for any surveillance system. For each of these challenges, we developed feasible solutions that are discussed.

**Conclusion:**

There are many challenges to overcome when implementing an electronic disease surveillance system, not only related to technology issues. A comprehensive approach is required for success, including: technical support, personnel management, effective training, and cultural sensitivity in order to assure the effective deployment of an electronic disease surveillance system.

## Background

Infectious disease surveillance systems provide epidemiological data that is essential to control, prevent and respond to diseases [1]. Electronic systems facilitate disease surveillance by reducing delays in data availability and usage and by improving data processing and outbreak recognition. However, implementing electronic surveillance is considerably more complex than simply applying new technology. There are a number of pertinent objectives that can be addressed by an electronic disease surveillance system, including: (1) detection of outbreaks of disease; (2) monitoring trends in rates of disease or effectiveness of an intervention; (3) strengthening the local capabilities in outbreak detection and response; (4) facilitating the generation of hypotheses by stakeholders that require further scientific investigation [2]. A minimum level of infrastructure and a critical number of trained personnel, as well as strong political support are needed. Most importantly, a surveillance-oriented culture is often completely absent and needs to be introduced, nurtured and reinforced. Finally, self-sustainability beyond the initial investments is difficult to attain and is limited by insufficient funding and competing (often more immediate) priorities within strained health systems [3,4].

In developing countries, epidemiologic surveillance is usually led by the Ministry of Health, and they often still use traditional pen and paper methods of disease reporting; however this approach is not efficient or practical for military populations in developing countries, since they are frequently deployed, dwell in remote areas, live in close quarters and share most of their activities and facilities; all of these factors leave them prone to rapid transmission of infectious diseases. A more suitable alternative was developed by investigators from the U.S. Naval Medical Research Center Detachment (NMRCD) in collaboration with the Peruvian Navy and Army. This initiative was based on Alerta, a near real-time electronic disease surveillance system that uses novel technologies and proven effective strategies affordable in resource-limited countries. The system collects reports on 45 clinical diagnoses or syndromes that correspond to infectious diseases of mandatory-notification or military relevance. Alerta's technology allows reporting via multiple methods: internet, toll free telephone access, and in remote sites, VHF radio relays to the regional hubs responsible for entering the data. Healthcare workers including physicians, nurses and technicians have been trained to operate the system [5].

Alerta was implemented in 2003 in the Peruvian Navy and currently covers 97.5% of the Navy population. It has grown from 11 to 88 sites reaching national coverage, and has proven to be useful in outbreak detection and establishing baseline disease incidence rates, as we detail below. Based on the successful experience with the Navy, the Peruvian Army decided to implement Alerta in June 2005, giving priority to their remote areas with endemic tropical diseases. Due to the previous experience with the Navy system, the Peruvian Army's expansion has been faster, and they currently receive reports from 120 units throughout the country. Many issues arose during the implementation process of these two systems, and solutions were devised and implemented promptly. The purpose of this paper is to identify and discuss these challenges and the best methods to address them.

## Methods

This is a retrospective description of the Alerta implementation process. The analysis of the system includes a description of each component: personnel, resources, and processes. Following this, we review the results of the system evaluation performed according to the CDC guidelines. Both steps allowed us to identify the main challenges we must overcome and the solutions applied in a developing country setting.

## Results

### System description

The Alerta electronic disease reporting system consists of three distinct components: personnel, processes, and resources. The stakeholders involved in the functioning of Alerta include the reporting unit personnel, the central hub personnel, and Naval and Army military authorities. The reporting unit personnel can be physicians, nurses, or technicians who have been trained in the functioning of the system before their deployments to the different Navy regions. The central hub has four physicians and two technicians who monitor and analyze the collected data. The authorities are high-ranking military officials who make medical policies based on the information provided by the central hub.

**Figure 1:**
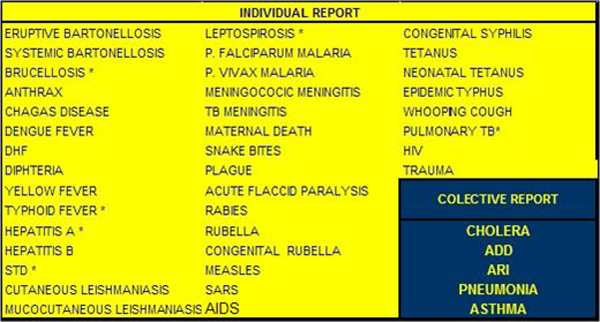
Diseases under surveillance by Alerta system.

**Figure 2:**
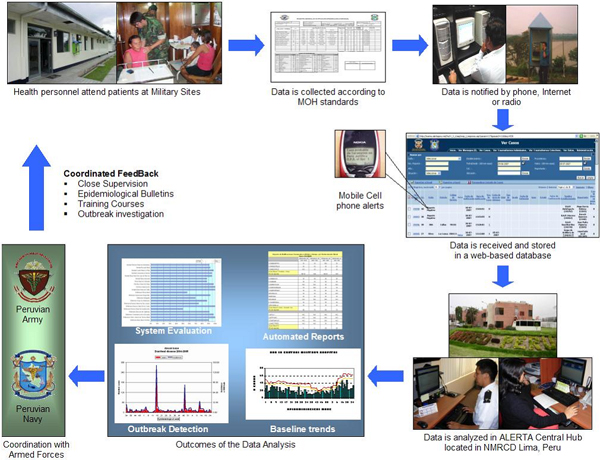
Flowchart of Alerta system.

The process of data collection begins with gathering of information at the reporting units by health personnel concerning diseases that are mandatory to report to the Peruvian Ministry of Health or relevant to the military (See Figure 1). A designated person proceeds with the notification process by summarizing the data and sending two types of reports: collective cases reported twice a week that include the most frequent syndromes (acute respiratory and acute diarrheal cases) and individual cases reported as soon as they are identified. Once the data are entered into the system, a monitoring process takes place, checking quality control and optimizing the database. The central hub can then analyze the data and look for indications of outbreaks or other trends. Processed information is returned to the stakeholders from each reporting unit as well as the authorities. The flowchart of the Alerta system is shown in Figure 2.

The system has no direct cost for the Peruvian Navy or Army because the system relies mostly on resources already in place. The resource requirements include the cost of the system itself, but also the trained health care personnel, the communications tools to be used to send reports, and the direct laboratory support in the case of an outbreak. There must be at least one trained person per site. These reporting personnel do not receive extra economic incentives, because their function in the system is part of their official duties. All the information is processed and analyzed by the central hub, located and financed by the U.S. NMRCD in Lima. Reports can be sent from any public phone in Peru via a toll free number or from a computer with internet connectivity. Additionally, the central hub provides training and support to all the stakeholders. The software used by Alerta is provided by Voxiva, a private company contracted by NMRCD, which represents the only direct cost of the system.

The product of the system is information in real time, which is displayed via the secure website or sent via automated reports to the cell phones of the stakeholders. This information has allowed us to establish baseline levels of the diseases under surveillance and to detect outbreaks. Finally the system can be self-evaluated in order to assess the accuracy and completeness of the data, and ultimately improve the functioning of the system.

### System evaluation

The system was assessed following the CDC guidelines for the evaluation of surveillance systems [1]. Since its implementation in January 2003 through December 2006, 18,878 reports (negative, collective and individual) have been received, containing 82,225 health-related events. The most frequent reported events were acute respiratory infections (ARI, 71.85%) and acute diarrheal disease (ADD, 24.30%); individual reports of other diseases were less frequent. The percentage of reports on time increased from 80% to 86% and the error rate diminished from 0.12 to 0.02 per report. The system identified 34 outbreaks during this period: 22 ADD, 4 Classic Dengue, 2 Malaria, 2 ARI, 1 each of conjunctivitis, scabies, rubella and mumps. According to the Navy and Army Health authorities, Alerta established baseline disease incidence rates for each reporting site, in many cases representing the first reliable facility-based data for remote regions [6].

### Identified challenges

Based on the implementation of Alerta and an evaluation of the system, we have identified 4 broad areas and 11 challenges to the implementation of the system, and propose the following solutions to address these issues.

### Stakeholders

#### Limited epidemiological training

There is limited experience with epidemiology and surveillance for infectious diseases within the health care system of the Peruvian military. The incorporation of a "notification culture" into the daily duties is an on-going challenge that has taken years to establish. All health personnel who are deployed to the different units are now trained in the basics of epidemiology, outbreak detection and the functioning of the surveillance system. This has clearly strengthened the local response to outbreaks and implementation of control measures.

#### Continuous movements of trained personnel

Health care personnel are frequently deployed to remote regions of the country, which often leads to a decrease in the reporting rate at sites that lose personnel. Training before deployments and local replication of the courses has created a cohort of personnel who can perpetuate the reporting process and maintain the system.

### Resources

#### Remote border area sites without access to phone and computers

The lack of access to phone or internet from very remote sites throughout Peru has hampered reporting not only for our populations but also for the Ministry of Health as well. We developed a Very Low Frequency (VHF) radio-relay process that reports through the nearest site with telephone or internet access.

#### Lack of laboratory confirmation of cases

The rate of confirmed cases remains low, approximately 40–50%, which limits the validity of syndromic surveillance. We have paired our surveillance sites with regional diagnostic laboratories where possible, and have streamlined the process of sending samples to our laboratory in Lima.

### Processes

#### Reporting a large number of cases

**Figure 3:**
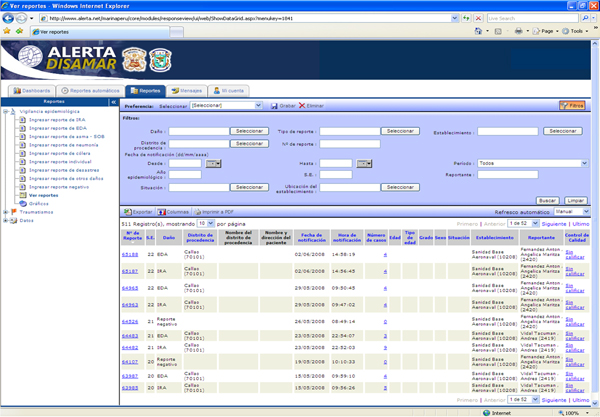
New platform of the Alerta system.

Some units were overwhelmed with large numbers of cases and had trouble reporting them on time. The use of standardized templates and reporting via electronics means solved this problem (Figure 3). The average time to enter a report into the system is now 2 minutes 40 seconds by phone and 2 minutes 16 seconds by internet.

#### Low report-on-time rate

The report on-time rate reached the desired level (85%) only last year, three years after the start of the system. For defining report on-time rate, we used all reports entered into the system by 14:00 hours each Monday (the weekly deadline) as on-time. Timeliness suffered particularly during the expansion phase of the system, when the number of trained personnel per site fell dramatically. The cohort of personnel at the reporting sites was trained and became accustomed to the system over the first two years, and time utilizing the system correlated directly with improved reporting on-time rates.

#### Data quality

Similar to the report on-time rate, the number of errors per report was higher in the initial implementation period and with the expansion of the system, especially when ships were incorporated last year. As the reporting sites became familiar with the system, the error rate decreased substantially from 0.12 to 0.02.

#### Outbreak detection and response

As the report on-time rate, the error rate and general epidemiologic knowledge have improved the number of outbreaks reported has increased. In the Navy, the system detected 2 outbreaks in 2003 and 12 outbreaks in 2007; each outbreak was reported on time (defined as within 24 hours of the significant increase in cases). In the Army, the system detected 9 outbreaks in 2007, with 8 of them reported on time. More importantly, the time to recognize an outbreak has diminished, making diagnosis and response much easier.

### Central hub

#### Data analysis within the system

The system incorporates data from many sites, and standard analysis often takes one week to perform. This standard analysis is now automated and consists of the assessment of report on-time rate, error per report rate, cumulative rates for each health event and the level of quality of the individual reports. The use of automated analysis and evaluation tools has allowed the stakeholders to generate graphics and tables at each reporting site (Figure 3), and to summarize the indicators for the principal health events reported to the system in a short period of time (eight hours on average).

#### Incorporation and maintenance of sites during the expansion

There were initially too many sites to include in the surveillance system, so choosing sentinel sites that were most at risk of infectious diseases or outbreaks was of paramount importance. Some sites, such as the ships and the border units, were very important to the Peruvian military due to preparedness. Continuous monitoring of the sites for timeliness and error rates allowed targeted training and adequate maintenance of the system.

#### Determining the effectiveness of the system

In developing settings, there are often not sufficient resources to pursue evaluations of surveillance systems. The CDC guidelines are a useful and standardized method to assess surveillance systems, and we have used these as well as developed new tools and indicators adapted to the local reality. Comparison with other systems in the region is also another important method of assessing effectiveness.

## Discussion

Currently, infectious diseases are the leading cause of morbidity and the second leading cause of death worldwide [7]. In spite of the advances in disease prevention and control, many public health challenges remain around the world. The growth of surveillance systems has made more data available and has increased the awareness to epidemic threats, but this evolution to more comprehensive and electronic systems has created many challenges [4,6]. Some of the new problems are cosmopolitan and others unique to developing countries. Through our work implementing a novel electronic surveillance system, we have acquired experience to effectively overcome many of these challenges in resource-limited settings. We believe that our approach could be useful for other public health officers and is important to generalize.

The first group of challenges is related to the stakeholders. The key to our solutions in this area relies on convincing the stakeholders that their efforts are useful and their valuable time is not wasted. In order to achieve this, we have attempted to change their approach to infectious diseases, using a broader perspective, that of epidemiology and public health. The idea was to create a "surveillance culture" and a sense that all participants are integral parts of the system. The best way to accomplish these goals was the extensive use of training, not only focused on the functioning of the system, but also on topics such as epidemiologic surveillance and outbreak response. As this knowledge was acquired by the reporters, the system was primed to receive more meaningful data and allow prompt response to outbreaks. Education seems to be the key solution for obstacles related to people.

The challenges related to resources require a different approach, particularly when financial support is scarce. The proposed solution is the use of the technology already in place to reduce costs. Instead of implementing all the system requirements at each site, we chose to adapt the system to the existing resources and use alternative and imaginative ways to report, such as the use of the VHF radio relay. Similarly, instead of building an expensive laboratory network, we decided to join efforts with different initiatives already in the region, avoiding the duplication of efforts. For instance, NMRCD's febrile disease surveillance and the influenza surveillance projects receive samples from the reporting units in order to confirm diagnoses, and the studies mutually benefit from increasing their sample size.

We based the critical analysis of our processes on the idea that public health informatics must ensure the most efficient way to convert information into action. Since technology allows managing large amounts of data in a short time, the first step was to adapt all our processes to the use of electronic tools. This meant that the stakeholders had to become accustomed to electronic devices, and assimilate more information more quickly, than with traditional systems. To help personnel in this task, training was mandatory but not sufficient; constant monitoring was also needed. One of our most successful strategies was to assign fully dedicated technicians to monitor all the sites and the data produced. This is particularly important during the expansion phase of any system, when most of the indicators were not optimized. Training and constant supervision strengthens local capabilities, and ultimately improves the response to public health threats.

Decision making is the main challenge at the central hub. Good quality data, sound analysis and options for action are all needed before an informed decision can be made. Automated analysis has proven to be an efficient way to perform complex assessments of the data by non-specialized personnel in short time periods [7]. The CDC guidelines to evaluate surveillance systems provide a comprehensive and structured method to address the attributes of any system, making it easier to interpret how well it is functioning. The availability of thorough information regarding not only the population under surveillance, but also the system itself, permit us to decide how to expand the system, which components need to be improved or dropped, and finally the long term goals and direction of our system.

## Conclusion

New tools to enhance infectious disease surveillance continue to be developed; optimizing their use is a necessity for public health. The experience with the Alerta system provides a valuable source of information on this issue, allowing us to generalize several lessons. It is very important to keep personnel interested through continuous training and supervision. Persistent and focused evaluation of the system is also required to assure optimal functioning of any surveillance system.

### Authors' contributions

GS participated in expansion and consolidation of the system and drafted the manuscript.  RA participated in the implementation; expansion and consolidation of the system and helped to draft the manuscript. JN participated in expansion and consolidation of the system and helped to draft the manuscript. MF Medical Director of the Health Care Department of the Peruvian Navy, provided the facilities to the development of the system. CL Medical Sub director of the Health Care Department for the Peruvian Army, provided the facilities to the development of the system. CC participated in the design, implementation, expansion and part of the consolidation of the system. DB participated in the implementation, expansion and consolidation of the system and helped to draft the manuscript.   

## Disclaimer

The views expressed in this article are those of the author and do not necessarily reflect the official policy or position of the Department of the Navy, Department of Defense, nor the U.S. Government. This work was supported by funding by the Navy work unit number 847705 82000 25 GB B0016. David L. Blazes is a military member. This work was prepared as part of his official duties. Title 17 U.S.C. §105 provides that 'Copyright protection under this title is not available for any work of the United States Government.' Title 17 U.S.C. §101 defines a U.S. Government work as a work prepared by a military service member or employee of the U.S. Government as part of that person's official duties.

## Competing interests

The authors declare that they have no competing interests.
